# 2589. *Legionella* pneumonia in cancer patients: 12-year experience at MD Anderson Cancer Center 2009-2021

**DOI:** 10.1093/ofid/ofad500.2204

**Published:** 2023-11-27

**Authors:** Takahiro Matsuo, Sebastian Wurster, Ying Jiang, Jeffrey Tarrand, Dimitrios P Kontoyiannis

**Affiliations:** The University of Texas MD Anderson Cancer Center, Houston, TX; The University of Texas MD Anderson Cancer Center, Houston, TX; The University of Texas MD Anderson Cancer Center, Houston, TX; The University of Texas MD Anderson Cancer Center, Houston, TX; The University of Texas MD Anderson Cancer Center, Houston, TX

## Abstract

**Background:**

*Legionella* pneumonia (LP) is an uncommon, yet severe pneumonia in cancer patients, but its clinical features are scarcely studied in contemporary cohorts. Therefore, this study aimed to characterize the clinical manifestations, therapy, and outcomes of LP in contemporary cancer patients.

**Methods:**

We identified cancer patients with LP at MD Anderson Cancer Center in 2009-2021. LP was diagnosed using *Legionella* urine antigen and/or culture from sputum, tracheal aspirate, or bronchoalveolar lavage. Species identification was achieved by Matrix-assisted laser desorption ionization–time-of-flight mass spectrometry or 16S rRNA. Independent predictors of 30-day mortality after LP diagnosis were determined using multivariable logistic regression analysis.

**Results:**

**Figure:**
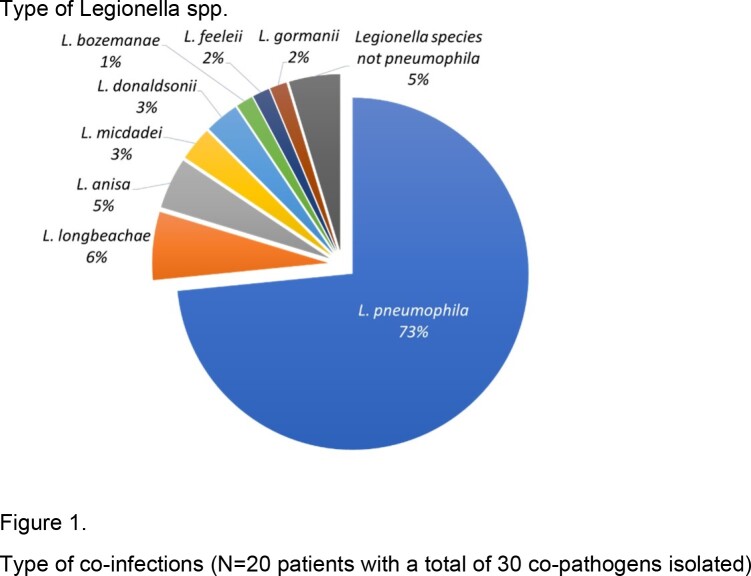

**Figure:**
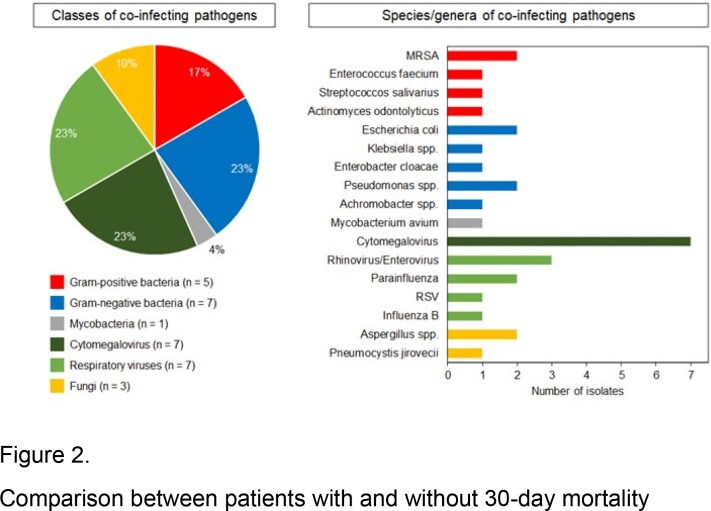

Table 1.

**Figure:**
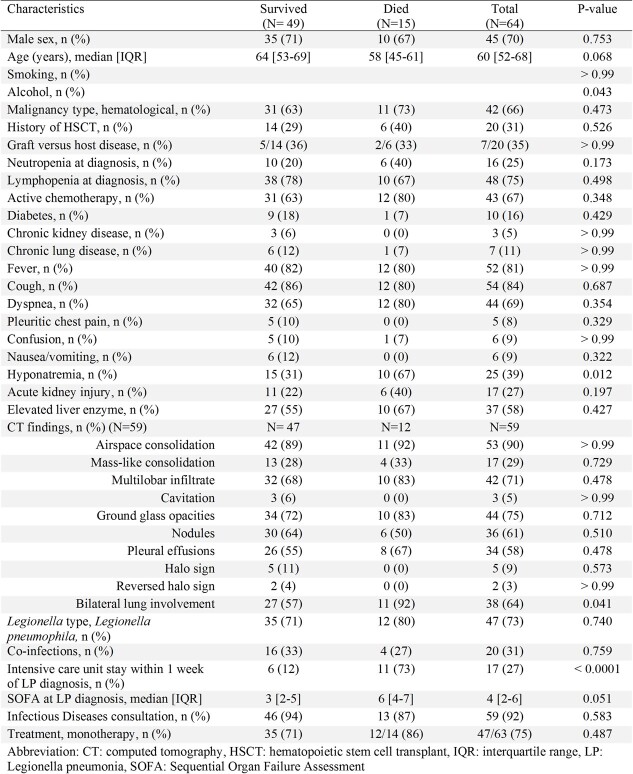

**Conclusion:**

We identified 64 LP patients; of which 47 (73%) were caused by Legionella pneumophila and 17 (27%) by Legionella non-pneumophila (Figure 1). Most patients (66%) had hematological malignancy, and 31% had a history of hematopoietic stem cell transplantation (HSCT). Lymphopenia (< 1000 cells/µl) was common (75%). Twenty patients (31%) had lung coinfections, especially those with a history of HSCT (9/20, 45%). A total of 30 co-pathogens were isolated, with Gram-negative bacteria, cytomegalovirus, and respiratory viruses (7/30, 23% each) being the most common co-pathogens (Figure 2). Twenty-five patients (39%) had hyponatremia and 17 (27%) had acute kidney injury. Most patients (64%) had bilateral lung involvement. All but one patient received appropriate antibiotics at the onset of LP. Thirty-day mortality of LP was 23% (Table 1). Independent predictors of 30-day mortality were hyponatremia (adjusted odds ratio [aOR], 3.03 [95% CI, 1.21-10.43], P = 0.013), bilateral lung involvement (aOR, 3.78 [95% CI, 1.16-28.98], P = 0.019), and Sequential Organ Failure Assessment (SOFA) score ≥5 (aOR, 2.76 [95% CI, 1.08-9.44], P = 0.029).

**Disclosures:**

**Dimitrios P. Kontoyiannis, MD, MS, ScD, PhD**, AbbVie: Board Member|Astellas: Grant/Research Support|Cidara: Board Member|Gilead: Grant/Research Support|Merck: Advisor/Consultant|Scynexis/MSGERC: Board Member

